# Separating two tightly linked species-defining phenotypes in *Bactrocera* with hybrid recombinant analysis

**DOI:** 10.1186/s12863-020-00936-1

**Published:** 2020-12-18

**Authors:** Heng Lin Yeap, Siu Fai Lee, Freya Robinson, Roslyn G. Mourant, John A. Sved, Marianne Frommer, Alexie Papanicolaou, Owain R. Edwards, John G. Oakeshott

**Affiliations:** 1 CSIRO Land and Water, Black Mountain, Canberra, ACT 2601 Australia; 2 Applied BioSciences, Macquarie University, Macquarie Park, Sydney, NSW 2109 Australia; 3 School of Biological, Earth and Environmental Sciences, University of New South Wales, Kensington, Sydney, NSW 2052 Australia; 4 Hawkesbury Institute for the Environment, Western Sydney University, Richmond, Sydney, NSW 2753 Australia

**Keywords:** *Bactrocera tryoni*, *Bactrocera neohumeralis*, Mating time, Callus colour, Hybridisation, Recombination, Selection, BACKCROSS

## Abstract

**Background:**

*Bactrocera tryoni* and *Bactrocera neohumeralis* mate asynchronously; the former mates exclusively around dusk while the latter mates during the day. The two species also differ in the colour of the post-pronotal lobe (callus), which is predominantly yellow in *B. tryoni* and brown in *B. neohumeralis.* We have examined the genetic relationship between the two characters in hybrids, backcrosses and multigeneration hybrid progeny.

**Results:**

Our analysis of the mating time of the parental species revealed that while *B. tryoni* mate exclusively at dusk, *B. neohumeralis* females pair with *B. neohumeralis* males during the day and with *B. tryoni* males at dusk. We found considerable variance in mating time and callus colour among hybrid backcross individuals of both sexes but there was a strong although not invariant trend for callus colour to co-segregate with mating time in both sexes. To genetically separate these two phenotypes we allowed the interspecific F1 hybrids to propagate for 25 generations (F25) without selection for mating time or callus colour, finding that the advanced hybrid population had moved towards *B. tryoni* phenotypes for both traits. Selection for day mating in replicate lines at F25 resulted in significant phenotypic shifts in both traits towards *B. neohumeralis* phenotypes in F26. However, we were unable to completely recover the mating time profile of *B. neohumeralis* and relaxation of selection for day mating led to a shift back towards dusk mating, but not yellow callus colour, by F35.

**Conclusion:**

We conclude that the inheritance of the two major species-defining traits is separable but tightly linked and involves more than one gene in each case. It also appears that laboratory conditions select for the *B. tryoni* phenotypes for mating time. We discuss our findings in relation to speciation theory and the likely effects of domestication during the generation of mass release strains for sterile insect control programmes.

## Background

Maintenance of species boundaries between hybridisable species in sympatry requires ongoing reproductive isolating mechanisms to impede interspecific gene flow. Since the inception of the Biological Species Concept [1], reproductive isolating mechanisms have been described in numerous sexually reproducing taxa. In addition to host shifts and pheromonal variation, temporal separation of potentially inter-fertile populations is another well-recognised premating barrier to reduce gene flow [2]. The timescale of such allochronic delimitation of life cycle events (e.g. reproductive activities) can range from different times of a day, to between seasons, or even between years [2].

In insects, daily allochrony in mating activities has been reported between sister species and within species in multiple lineages. For example, two sister species of spodopteran moths (*S. latifascia* and *S. descoinsi*) copulate at different times at night, as do two host strains of *S. frugiperda* [3, 4]. In *Anopheles* mosquitoes*,* incipient species *An. gambiae* and *An. coluzzii* form mating swarms at different times after sunset [5]. In tephritid fruit flies, intraspecific variation in reproductive timing has evolved in *Anastrepha fraterculus* where geographically separated ecotypes have mating receptivity times a few hours apart [6]. Variation in mating time within tephritid species can also be artificially induced in the laboratory over time, through selection for different developmental time, e.g. in *Zeugodacus cucurbitae* [7] or via long term laboratory domestication, e.g. in *Bactrocera oleae* [8].

Two other tephritids, the Queensland fruit fly, *Bactrocera tryoni*, and its sibling species the lesser Queensland fruit fly, *Bactrocera neohumeralis*, offer a unique model system to investigate the genetic basis of mating time variation. They are taxonomically placed in the same *tryoni* species complex and the only taxonomic feature used to separate the two species is the callus (post-pronotal lobe) colour (yellow in *B. tryoni*, brown in *B. neohumeralis*) [9]. *B. tryoni* is currently the major horticultural pest in Australia [10] and is widespread throughout the Australian eastern seaboard [11]. *B. neohumeralis* is a lesser pest largely restricted to the coastal region of Queensland and northern New South Wales, Australia, so its range is almost completely nested within that of *B. tryoni* [12]. The two species also overlap extensively in their fruit hosts [9], and produce similar amides in the male rectal glands, which are thought to be the pheromone production sites [13]. Despite the absence of geographic, ecological and pheromonal difference and post-zygotic barriers, they remain “good” species. The only known reproductive isolating mechanism is their distinct mating times: currently available data show *B. tryoni* is strictly a dusk-mater [14, 15] while *B. neohumeralis* mates in the morning to late afternoon [16].

Natural occurrences of intermediate callus colours have raised questions as to whether hybridisation could occur in the wild [17–25]. The existence of natural hybridisation is suggested by the similarity between the wild flies with intermediate callus colour and those hybrids obtained through interspecific crosses in the laboratory [25], but the high levels of intraspecific phenotypic plasticity in callus colour casts doubt on such inferences [19, 20]. Genetic comparisons based on microsatellite markers have found that individuals with higher amounts of yellow on their calli are always *B. tryoni* and led the authors to conclude that true genetic intermediates do not exist in the wild [26]. On the other hand, the presence of shared polymorphisms and haplotypes in two nuclear (*white* and *ITS2*) and two mitochondrial (*cytb* and *COII*) loci between *B. tryoni* and *B. neohumeralis* would at least be consistent with some hybridisation [27].

Although it remains unclear if natural hybridisation occurs or has occurred, hybridisation can be readily induced in the laboratory and is amenable to classical genetic analyses [16, 23–25, 28, 29]. Previous studies have established that *B. tryoni* mating time and callus colour are at least partially dominant in the F1 hybrids, and each trait is controlled by multiple autosomal genes [16, 28]. Meats et al. [30] also showed via selection in early generation hybrids that the two traits are linked, i.e., selection for yellow calli generates more dusk maters. The hybrid phenotypes appear to be unstable over multiple generations in the laboratory, as they tend generally towards *B. tryoni* callus colour if hybrids are kept independent of parental populations [18, 24]. While male mating behaviours are relatively well documented, the behaviours of the females have not been systematically explored at the genetic level. Furthermore, although evidence for some genetic linkage between callus colour and mating time is unequivocal, the nature of the association between the characters and its fate over multiple generations following interspecific crossing remains poorly understood.

The genetics of mating time is also directly relevant to the control of *B. tryoni* populations in the field, insomuch as the phenotype is key to the success of Sterile Insect Technique (SIT) programmes currently being implemented and scaled up in Australia [31]. It is critical that the mating times of the sterile males released in these programmes are synchronous with those of the wild flies whose mating they are intended to disrupt, and there is already evidence that mating time can shift significantly in the mass-reared flies (e.g. *B. oleae* [8]). The ability to hybridise *B. tryoni* and *B. neohumeralis* and monitor the phenotype over generations in the hybrids provides an ideal model system to dissect the genetic basis of the trait.

Here we report the results of two sets of genetic experiments to address these knowledge gaps. In the first we monitored the two traits (mating time and callus colour) in offspring of F1 hybrids backcrossed with *B. neohumeralis* males. Female informative backcrosses with *B. neohumeralis* males were used because meiotic recombination is limited to females and the two *B. neohumeralis* traits are recessive to those of *B. tryoni* [28, 30]. In contrast to previous studies [16, 28–30], we observed each mating time separately for each sex and mated the hybrids to parentals only. In the second experiment a population set up from F1 hybrids was left for 25 generations without deliberate selection for either character, and then selected for early mating for two generations, finally reverting to the regime without deliberate selection for a further eight generations. Both characters were scored at intervals across the 35 generations. Together these experiments provided a rigorous, sex-specific, investigation of the genetic architectures of the two traits and relationships between them.

## Results

### Genetic architecture of mating time variation and callus colour based on backcross analysis

**Fig. 1:**
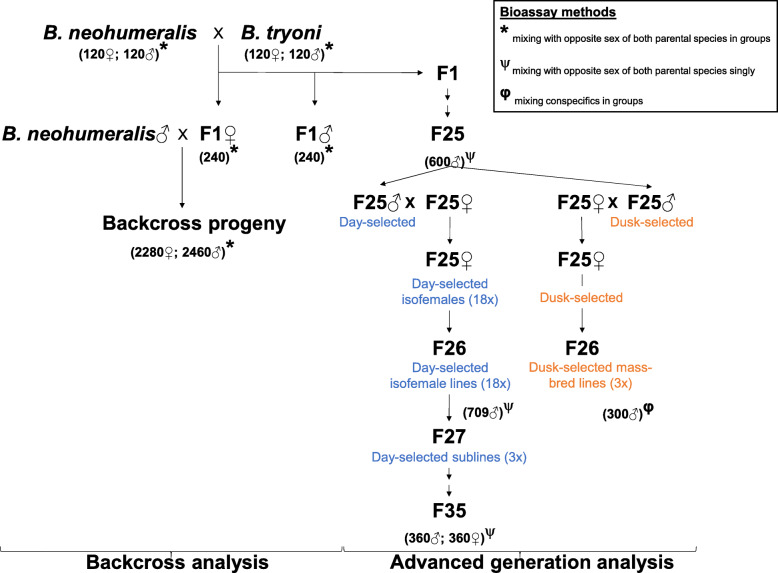
Crossing scheme illustrating the relationships of key bioassayed generations. Three methods were used to assay mating time in the backcross and the advanced generations. Numbers of individuals bioassayed are shown in brackets under each parental species, various generations (F1, backcross progeny, F25, F26 and F35) and the day- or dusk-selected lines. Note: These numbers do not represent the replicate structure, or the numbers of individuals used in the crosses (see Material and Methods for details)

**Fig. 2:**
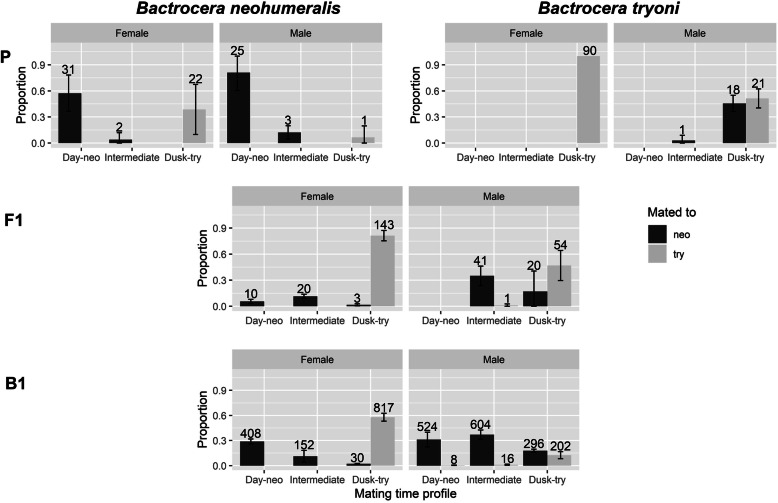
A backcross analysis of mating time profile. P: parental *B. neohumeralis* and *B. tryoni*; F1: F1 hybrids; B1: offspring of F1 female x *B. neohumeralis* male. Numbers above bar are the total number of observed mating pairs across three replicate lines. The average proportions were calculated separately for each line by sex, with the confidence interval of the proportion across the three replicates included on each bar

Analysis of a backcross population not only confirmed the phenotypic divergence in the two parental species and dominance relationship of the phenotypes, but also revealed interspecific differences in females, linkage between traits and the relative genetic complexity of each trait. These observations are shown in Figs. 1 and 2. In the parental generation (P), *B. tryoni* males primarily mated at dusk (under our conditions, the hour of < 100 lx between day and night; see Material and Methods) and *B. neohumeralis* during the day (at least 3 h before dusk) (39/40 or 97.5% vs 25/29 or 86% respectively). While female *B. tryoni* mated exclusively at dusk, female *B. neohumeralis* mated both during the day (with *B. neohumeralis* males) and at dusk (with *B. tryoni* males). Thus, amongst the mates of *B. tryoni* males at dusk, approximately half (18/39 or 46.2%) were *B. neohumeralis,* suggesting that *B. tryoni* males did not distinguish between the two types of females, and that females of both species could mate at dusk. The interspecific difference in female mating behaviours prompted the inclusion of virgins from both parental species in subsequent mating bioassays, to ensure adequate supply of suitable mates.

Although not quantified formally in the present study, we observed numerous indiscriminate mating attempts by *B. neohumeralis* males on both *B. tryoni* and *B. neohumeralis* females during the day. However, these mating attempts by *B. neohumeralis* males resulted in conspecific pairings only, despite the presence of *B. tryoni* females (Fig. 2). We also observed *B. neohumeralis* males attempting to copulate with *B. tryoni* females during the day in the absence of *B. neohumeralis* females; again, none of these attempts were successful (see *B. tryoni* females in Fig. 2). These observations imply that *B. tryoni* females were unwilling to mate during the day rather than the lack of courtship effort by the *B. neohumeralis* males.

F1 hybrid males were largely dusk maters (Fig. 2), which suggested that dusk mating was the more dominant trait. However, the dominance of dusk mating was not absolute because a significant minority (42/116 or 36.2%) of matings also occurred one to 2 h before the onset of dusk. A similar dominance relationship was also detected in the female F1 hybrids, where dusk mating was the most abundant class (146/176 or 82.9%), but unlike the F1 males, a few instances (10/176 or 5.7%) of day mating as defined above were observed. The phenotypic distributions of the F1 hybrids indicated that dusk mating was dominant in both sexes and that the level of dominance was more pronounced in males.

Relative to F1, the backcross progeny (B1) had a wider phenotypic distribution and a higher abundance of day maters in both males (F1 vs B1, percentile based bootstrap 95% confidence interval of median: + 0.50 h vs − 1.55 to − 1.89 h) and females (F1 vs B1, percentile based bootstrap 95% confidence interval of median, + 0.50 h vs + 0.37 to + 0.45 h) (Fig. 2). Such phenotypic shifts in mating time were consistent with the direction of the backcross (i.e., to male *B. neohumeralis*). B1 males mated predominantly with *B. neohumeralis* during the day and *B. tryoni* at dusk, in accordance with the mating profiles of the parentals. The polygenetic nature of this trait was supported by the high level of phenotypic variance in the B1 progeny of both sexes, although major gene effects were implied by the bimodality of the distribution for females. Notably the bell-shaped phenotypic distribution in B1 males reflected a more complex genetic control of mating time (i.e., more genes involved) in that sex (Fig. 2).

**Fig. 3:**
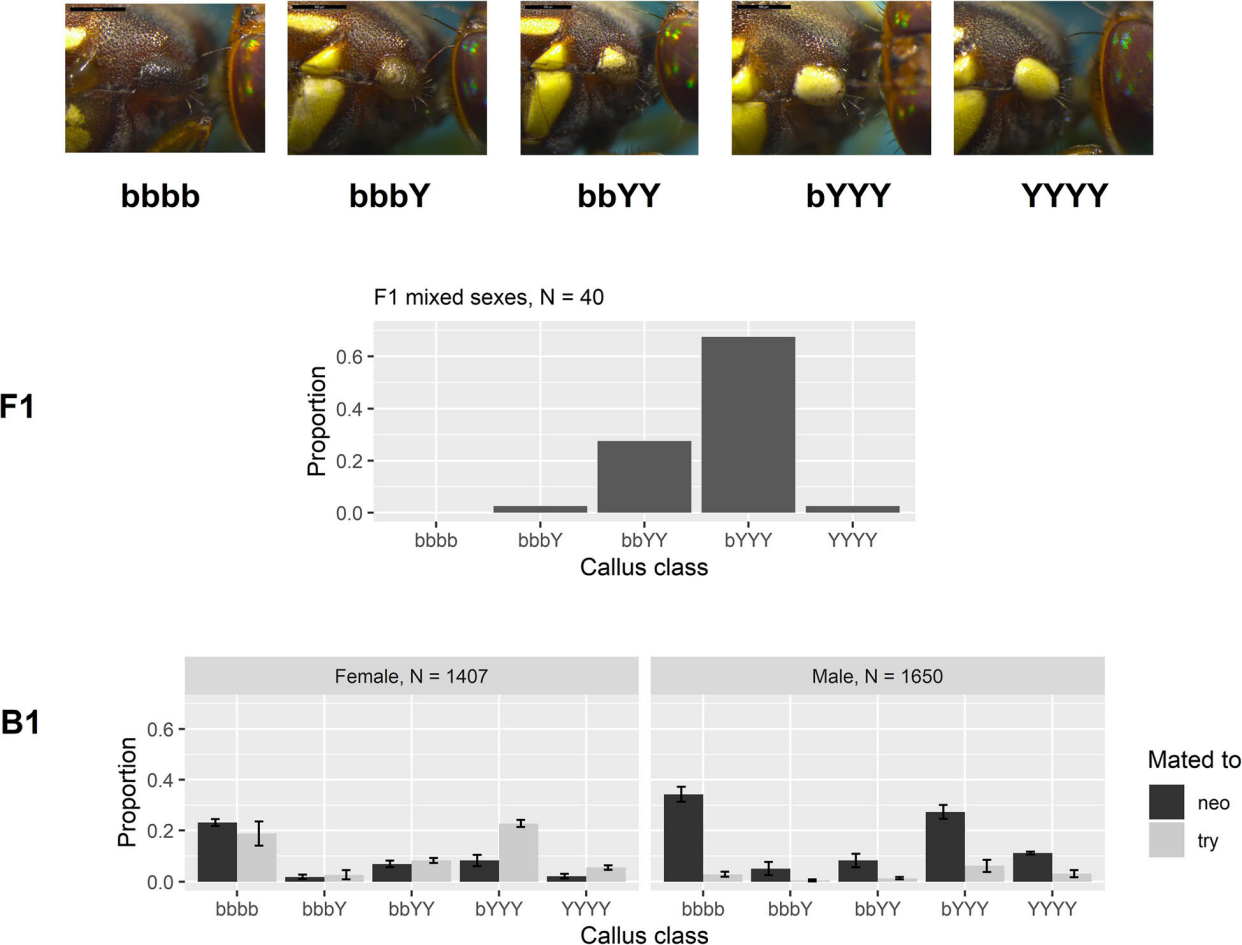
A backcross analysis of callus colour profile. Callus colour was grouped into five classes in ascending order of yellow in the callus: bbbb, bbbY, bbYY, bYYY and YYYY. *B. neohumeralis* parentals were almost always ‘bbbb’ while *B. tryoni* parentals were ‘YYYY’. Row F1shows the callus colour distribution in F1 hybrids, while row B1shows the callus colour distribution in the F1 x *B. neohumeralis* backcross partitioned by the sex and species of parental mated to the B1 individual. The average proportions were calculated partitioning by sex with the standard deviation of the proportion across the three replicate lines included on each bar

Callus colour was grouped into five ordinal categories, ranked in ascending order of yellowness on the callus as illustrated in Fig. 3. The five groups were: “bbbb”, completely brown (i.e., the *B. neohumeralis*-like callus); “bbbY”, traces of yellow (≤ 25% yellow); “bbYY”, approximately 50% yellow; “bYYY”, > 70% yellow; and “YYYY”, completely yellow (i.e., the *B. tryoni*-like callus) (Fig. 3). The F1 phenotypic distribution was heavily skewed towards yellow, with 39/40 (or 97.5%) individuals in the callus colour group “bbYY” or above (Fig. 3). The results indicated that yellow callus was dominant to brown callus, but the dominance was not absolute because most F1 individuals fell within the intermediate and yellow-leaning categories (“bbYY” (27.5%) and “bYYY” (67.5%) respectively). Compared to the F1 generation, the B1 progeny exhibited higher phenotypic variation, encompassing both phenotypic extremes (39.2% “bbbb” and 11.1% “YYYY”), along with all other intermediate categories (Fig. 3). Furthermore, a bimodal distribution around “bbbb” and “bYYY” was evident in both male and female B1 progeny, suggesting the presence of a major effect locus and several minor effect genes (Fig. 3).

### Mating time and callus colour are genetically linked

**Fig. 4:**
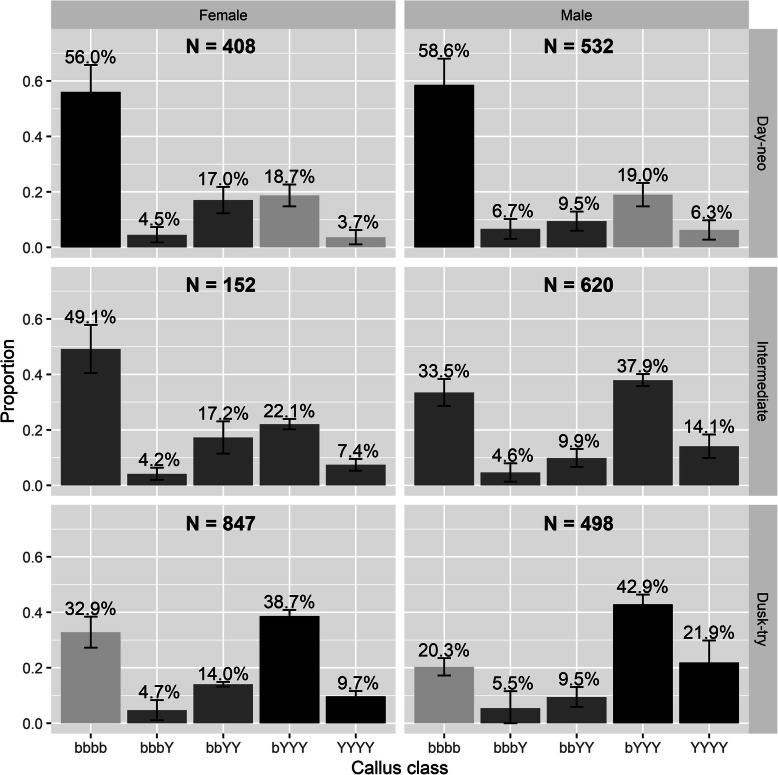
Callus colour profile partitioned by sex and the three mating time classes. Percentages above bar are the average percentage of observed in each callus colour class across three replicate lines. The average proportions were calculated partitioning by sex and day/dusk mating with the standard deviation of the proportion across the three replicate lines included on each bar. Black bars represented parental-like phenotypes (day mating brown callus, dusk mating yellow callus); light grey bars represented recombinant-like phenotypes (day mating yellow callus, dusk mating brown callus); and dark grey bars represented intermediate phenotypes for callus colour or mating time or both

Analysis of the B1 generation indicated that mating time and callus colour were genetically linked. Thus, female and male day maters were 56.0 and 58.6% bbbb (i.e., parentals) respectively, whereas female and male dusk maters were only 32.9 and 20.3% bbbb (i.e., recombinants) respectively (χ^2^ > 87, df = 4, *P* < 0.001) for both males and females; Fig. 4). Conversely, female and male dusk maters were 9.7 and 21.9% YYYY (i.e., parentals) and 38.7 and 42.9% bYYY (i.e., parental-like) respectively, whereas female and male day maters were only 3.7 and 6.3% YYYY (i.e., parentals) and 18.7 and 19.0% bYYY (i.e., parental-like) respectively.

We interpret the fact that the parental-like bYYYs outnumbered the parental YYYYs in the latter comparison as indicating that a major gene for yellow callus linked to the major callus colour gene was sufficient to bestow a bYYY phenotype, with other, largely unlinked, genes of minor effects also required for a full YYYY phenotype.

### Male mating time trended towards dusk but remained tightly linked to callus colour in F25

**Fig. 5:**
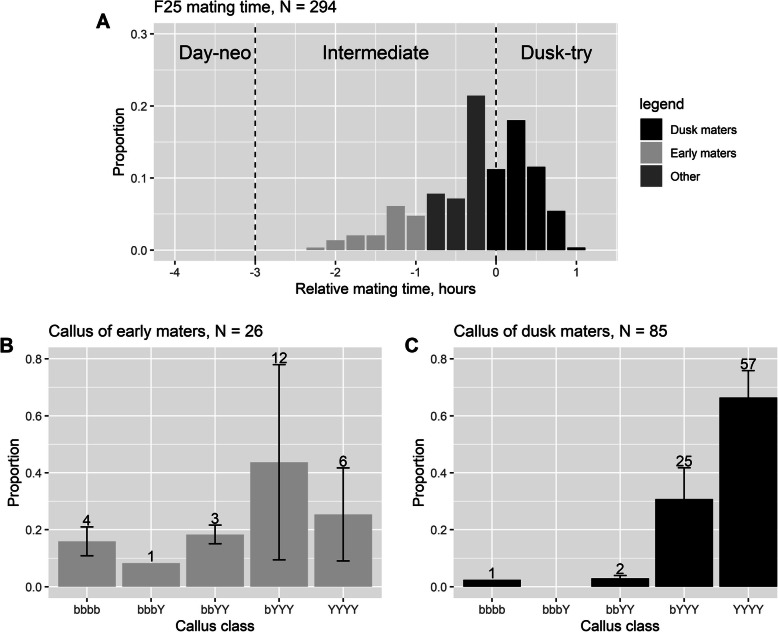
Male mating time and callus colour profiles of F25 hybrids. **a** Mating time profile of F25 males. Relative mating time is the number of hours from the onset of dusk, where negative values are before dusk. Black bars represented dusk mating and light grey bars early mating; (**b**) callus colours amongst the early mating F25 males; (**c**) callus colours amongst dusk mating F25 males. Numbers above the bars are the total number of individuals observed in each callus colour class across three replicate lines. The proportions were averaged across three replicate lines and the standard deviation of the proportion across the three replicate lines was included on each bar

Mating time of male F25 hybrids spanned ~ 3 h, from ~ 2 h before dusk to ~ 1 h after dusk; none of the 294 males tested mated in the strictly day mating phase, i.e., > 3 h before dusk (Day-neo in Fig. 5a). The overall phenotypic distribution of the F25 generation was similar to that in the F1s (Fig. 2). Although the proportion of day maters in F25 should be low given the dominance relationship, the complete absence of day mating males in 294 F25 individuals suggests that day mating had been selected against in the laboratory.

As with mating time, callus colour at F25 had trended towards the *B. tryoni* phenotype, i.e., yellow (Fig. 5b and c). However, the trend was weaker for callus colour than for mating time, as indicated by the presence of some males with *B. neohumeralis*, i.e., brown, calli (e.g., four of the 26 early maters and one of the 85 dusk maters).

In fact, the callus colour profiles between the early maters and dusk maters were markedly different: early maters had a more evenly distributed callus colour range whereas the dusk maters skewed heavily towards yellow (bYYY and YYYY) (Fig. 5b and c), and contingency test indicated that callus colour and mating time were still not independent (χ^2^ = 24.381, df = 4, *P* < 0.001), which can be interpreted as persistent linkage between the two.

Overall, we interpret the F25 data as indicating that the major loci controlling the two traits were tightly linked but could be de-coupled and that selection for dusk mating had been operating in the intervening generations. The weaker trend towards yellow callus colour at F25 could be due to independent selection for this phenotype or hitchhiking on the selection for dusk mating.

### Selection for early mating time generated different mating profiles in different isofemale lines

The persistence of the association between mating time and callus colour at F25, together with the strong trend towards *B. tryoni* phenotypes, prompted a series of selection experiments to investigate how these traits respond to artificial selection. F25 males were selected for early mating (at least 1 h before dusk) and dusk mating. A significant difference was detected among the F26 progeny of the 18 isofemale lines selected for early mating (Kruskal-Wallis χ^2^ = 86.5, df = 17, *P* < 0.001), which confirms the polygenic nature and genetic complexity of the phenotype. Selection for dusk mating at F25 did not alter the mating profile significantly in any of the three dusk-selected populations at F26, perhaps because they were heavily skewed towards dusk mating already.

### Callus colour co-segregated with mating time after a single round of mating time selection

Lines that were selected for male early mating in F25 had significantly higher proportion of brown-leaning callus colours (classes bbbb and bbbY) and intermediate (class bbYY); and a lower proportion of yellow callus (bYYY and YYYY) when compared to unselected lines in F26 (Table 1). The opposite was seen in lines selected for male dusk mating vs unselected lines (Table 1). The co-segregation of mating time and callus colour in these selected lines provided additional evidence for strong genetic linkage of the major loci underlying the two traits.

**Table 1:** Mean proportion of each callus class with its 95% confidence interval in F26 lines that were selected for early mating or dusk mating, or unselected. Pairwise *t*-tests with unequal sample size were performed for each callus class testing the differences of early mating selected vs unselected and dusk mating selected vs unselected

	Early mating selected		Unselected line		Dusk mating selected
Callus	Mean, 95% CI	Test1	Mean, 95% CI	Test 2	Mean, 95% CI
bbbb	0.082 ± 0.025	**	0.020 ± 0.022	NS	0.003 ± 0.006
bbbY	0.155 ± 0.074	**	0.028 ± 0.020	NS	0.011 ± 0.007
bbYY	0.088 ± 0.036	*	0.035 ± 0.013	*	0.011 ± 0.010
bYYY/YYYY	0.675 ± 0.095	***	0.917 ± 0.040	*	0.975 ± 0.016

Test 1 is the comparison between early mating selected and unselected lines. Test 2 is the comparison between dusk mating selected and unselected lines

*NS* Non-significant; * 0.01 < *P* ≤ 0.05; ** 0.001 < *P* ≤ 0.01; *** *P* ≤ 0.001

Callus classes bYYY and YYYY were combined because frozen material was used in which these two categories could not be distinguished reliably

### Consistent responses to selection and relaxation of selection for early mating among sublines across multiple generations

**Fig. 6:**
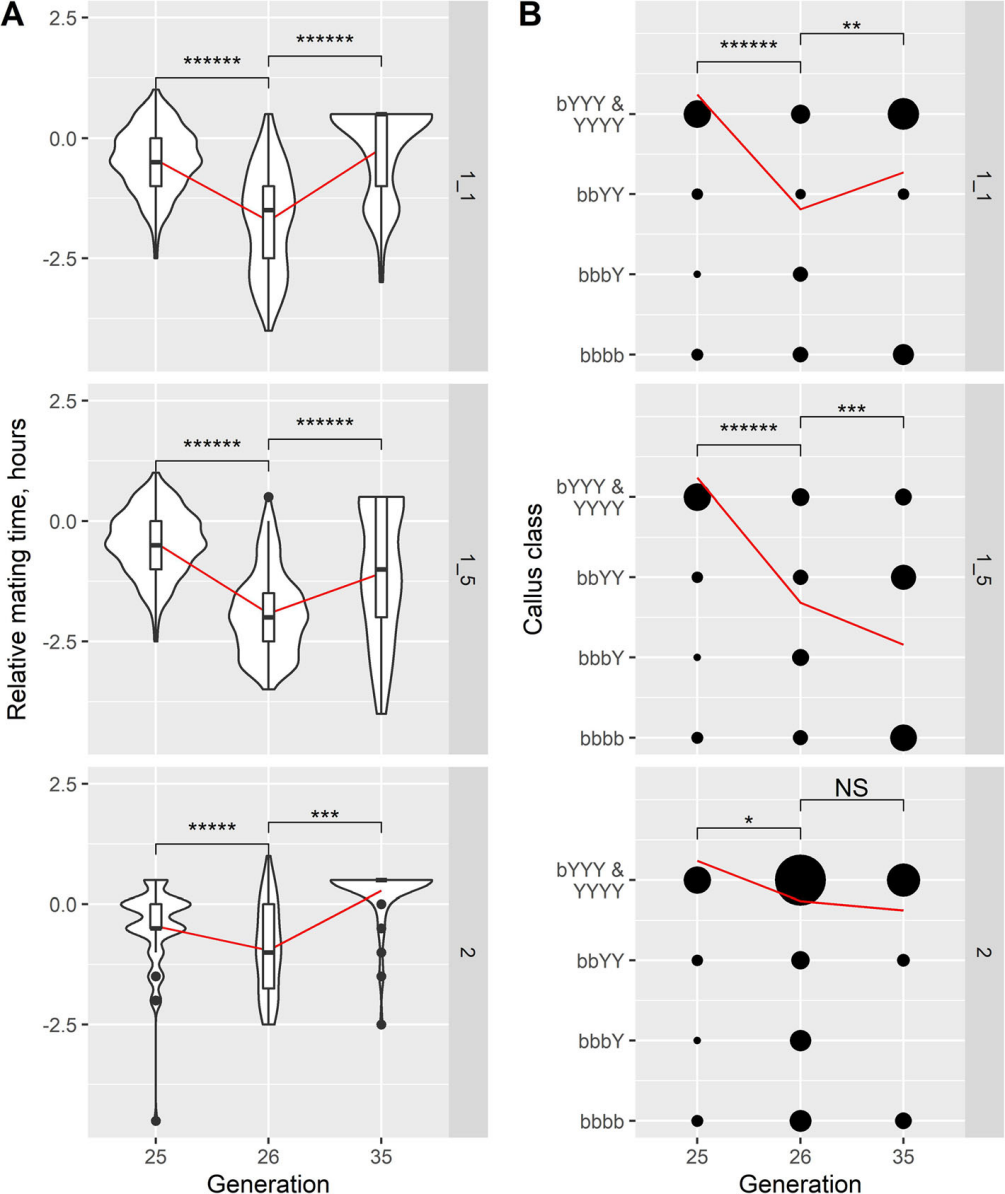
Phenotypic responses to selection for early mating and relaxation of selection in advanced generation hybrids. **a** Violin plot, box plot and line plot of mean from generations 25, 26 and 35 for mating time partitioned by the three lines maintained after selection for early maters. A trendline (red line) for the mean mating time was plotted for each subline across generations. **b** Bubble plot of callus colour in generations 25, 26 and 35, partitioned by the three lines maintained after selection for early maters. Sample size is proportional to the size of the bubble. A trendline (red line) for the mean of the ordinal callus classes was plotted for each subline across generations. F25 represented the pre-selection generation; F26 represented the post-1st selection generation while F35 was eight generations of no selection after selection was applied at F25 and F26. Pairwise Mann-Whitney *U* tests were performed on the F25-F26 and F26-F35 comparisons

After selection for early mating (at least 1 h before dusk) in males at F25 and F26, three sublines were propagated for a further eight generations without deliberate selection on either phenotype or progeny re-scored at F35 (Fig. 6a). Two sublines were isofemale lines while the third was a pool of seven isofemale lines. Across the three sublines, there were just 7.33% (44/600) early maters in F25 (i.e., pre-selection), followed by an increase to 63.0% (447/709) in F26 (i.e., one generation after selection) but a reduction to 18.3% (66/360) in F35. All three sublines showed similar trends: Thus, there was a shift in average mating time away from dusk towards early day between F25 and F26 followed by a reversal to near dusk in F35 (Fig. 6a). These results showed that mating time was responsive to directional selection (i.e., early mating in this case) but had the tendency to shift back to dusk mating (*B. tryoni*-like phenotype) in the absence of selection.

### Selection for early mating increased frequency of brown callus class but relaxation of selection yielded inconsistent trends

Selection for early mating at F25 was accompanied by a significant collateral shift in callus colour distribution from the two most yellow classes (bYYY and YYYY) to lower levels of yellow in all three sublines (Fig. 6b). In the absence of selection however, the sublines produced different trajectories between F26 and F35, where one subline (1_1) showed increased in yellow-leaning callus classes, one increased (1_5) significantly for brown-leaning callus while subline 2 had no significant change (Fig. 6b). This suggests that callus colour was not under directional selection in the laboratory and was no longer consistently hitch-hiking on the changes in mating time either.

### The frequency of recombinants increases in one subline after selection for early mating

Our totals of 137 F25 and 131 F35 dusk mating males included 27 and 36 respectively which were found to mate at dusk consistently on two successive days of testing. Amongst these subsamples of dusk maters whose phenotype was strongest/most reliable we found one (i.e., 3.7%) and five (i.e., 13.9%) that had brown calli, i.e., were recombinants. The five at F35 comprised one of 14 (7.1%) in subline 1_1, four of five (80%) in subline 1_5 and none of 17 in subline 2. The percentages for three F35 sublines were significantly different from each other (χ^2^ = 21.55, df = 2, *P* < 0.001) and the 80% for subline 1_5 was clearly much higher than the 3.7% for F25 dusk maters (Two-sample test for equality of proportions with continuity correction, χ^2^ = 13.29, df = 1, *P* < 0.001). However, the other two sublines were not significantly different from F25. The enrichment of recombinants in at least one selected inbred line confirms that the major loci controlling these two phenotypes are distinct yet genetically separable, despite strong linkage. The significant heterogeneity between the sublines likely reflects founding effects in the establishment of the isofemale lines.

## Discussion

Our analysis of mating patterns among the parental species show that *B. tryoni* males and females both strongly prefer to mate at dusk and conversely that *B. neohumeralis* males prefer to mate during the day (i.e., at least 3 h earlier than dusk). Crucially however we find that *B. neohumeralis* females will mate at dusk as well as during the day and that *B. tryoni* males show little preference for conspecifics over *B. neohumeralis* females at dusk. Thus, we can say that mating time differences between the species are not the same in the two sexes and that, beyond their mating time preference, *B. tryoni* males at least show no additional behavioural preference for conspecific females.

Consistent with an earlier report [28], we find that dusk mating in F1 hybrids behaves as an incompletely dominant character in both sexes, as does yellow callus colour, albeit the dominance in the former is slightly more pronounced in females than males. The progeny of backcrosses to *B. neohumeralis* (bearing the recessive phenotypes) generated relatively broad phenotypic distributions for both characters in both sexes, indicating some level of polygenic control. However, the distributions for female mating time and the callus colour of both sexes were still bimodal, suggesting some relatively simple major gene effects, whereas the bell-shaped distribution of mating time in males suggests a more complex genetic basis.

Our results also provide evidence for an association between callus colour and male-female mating time in B1: yellow callus females were more likely to mate with *B. tryoni* males while brown callus females behaved similarly to *B. neohumeralis* females (i.e. could be mated to *B. neohumeralis* during the day and to *B. tryoni* males at dusk). This association was not observed in males; there was a higher proportion of mating to *B. neohumeralis* females across all male callus colour classes. The discordance in mating suggests that males (at least in *B. tryoni*) do not mate assortatively.

Notwithstanding the two significant complexities in the mating time data (i.e., little discrimination by *B. neohumeralis* females and polygenic inheritance, particularly in males), the two characters showed significant linkage in the backcross progeny, in both sexes. However, recombinant phenotypes of both types were found, and in both sexes (Fig. 4, see light grey bars). This discounts one possible scenario, namely that the two characters are encoded by the same genes.

After 25 generations without deliberate selection in either direction for either character our hybrid population had evolved towards the *B. tryoni* state for both characters, although the trend was notably stronger in respect of mating time than callus colour. No day maters of either sexes were found at this point and relatively few intermediate mating times were evident either. By contrast some individuals with the brown callus colours characteristic of *B. neohumeralis* were recovered, as were several individuals with intermediate callus colouration. These results suggest our laboratory conditions selected strongly for dusk mating. However, we are unable to discriminate whether the callus colour changes are based on independent but somewhat weaker selection for the *B. tryoni* phenotype and/or hitchhiking effects based on the linkage with mating time genes.

On one hand, some level of hitchhiking would be expected, given the genetic linkage evident in the backcross generation and that there was still a strong association between the two characters at generation 25. On the other hand, it is perhaps surprising that the linkage between the two characters was still detectable after 24 generations in which recombination could have occurred. Out of 85 dusk mating F25 males, 57 had yellow callus (“YYYY”) and 25 mostly yellow callus (“bYYY”) but only one had brown callus (“bbbb”). In the absence of selection at least, linkage disequilibrium (LD) should decay at a rate of 1 – (1 – θ)^*t*^ per generation, where θ is the recombination frequency and *t* is the generation time. Given the level of genetic linkage (15% recombinant or θ = 0.15) detected in the backcross generation and the predicted LD decay (98.3% after 24 generations), the unexpected persistence of linkage between two phenotypes at F25 might suggest that some selection involving callus colour, either independent of mating time or epistatically associated with it, may have been operating, in addition to any hitchhiking.

One factor which could have contributed to the persistence of the linkage could be the accumulation of nucleotide differences and/or chromosomal rearrangements between the species that are mildly incompatible in the hybrids [32]. It is unclear how much genomic incompatibility exists between the two species, although it seems unlikely that their genomes are completely syntenous. Even small chromosome inversions encompassing major genes controlling either or both traits could not only disrupt hybrid fitness but also substantially reduce recombination involving those genes. Such effects, combined with potential hitchhiking between mating time and callus colour, and their dominance relationships (Fig. 2), might therefore explain the prevalence of the *B. tryoni* phenotypes in the advanced generations.

Deliberate selection for early mating time at generations 25 and 26 did not yield any full day matings (i.e., > 3 h before dusk) but did shift the distribution of mating times somewhat towards earlier mating. It appears that some of the genes contributing to day mating were still segregating in the population, but others may have been lost. Although the shift in mating time was relatively small, it still resulted in a shift towards *B. neohumeralis* callus colour as well. In combination with all the other information above, this suggests either very strong linkage between some genes contributing to the two characters, or very strong epistatic selection, or both.

The data from generation 35, i.e., after eight generations without deliberate selection, concurred with the generation 25 data in showing an overall movement towards the *B. tryoni* state for mating time. However, two aspects of the generation 35 data also provided additional insight. First, the frequency of recombinants had increased significantly in one instance. Second, while all three sublines had moved towards the *B. tryoni* mating time, only one did so for callus colour. These results suggest that the two characters could indeed be substantially separated given sufficient time.

Our findings present an intriguing scenario where the “dusk mating genotype” simultaneously suppresses day mating in both sexes, limits mating time to dusk, triggers courtship behaviours in males and activates female receptivity, and restricts these sexual interactions to a narrow window around dusk. It is difficult to envisage a single gene controlling all these complex phenomena and indeed we found it was polygenically inherited. However, the bimodal distribution of the phenotype in backcross females and the tight association with callus colour still implies some major gene effect. Paradoxically, it would therefore seem that the ‘major gene’ linked to callus colour entrains several different aspects of phenotype mediated by other genes in various pathways.

The limited prior work bearing on the mechanism behind the mating behaviour phenotypes bears out the complexity. Thus early work demonstrated that mating activity is responsive to both the circadian clock and light intensity and that the two responses are independently controlled [16] and a subsequent locomotor study showed that *B. neohumeralis* was consistently active throughout daylight hours whereas *B. tryoni* was less active through most of the day but had a peak of activity around dusk [33]. The latter study also found that *B. tryoni* had more brain transcripts that were differentially regulated between night and day than did *B. neohumeralis*. Further quantifiable experiments examining individual components leading up to mating (male courtship initiation, female mating receptivity, circadian entrainment in both species) are needed to disentangle the mechanistic complexities of this phenotype.

In sum, we conclude both characters differentiating the two species, particularly mating time, are polygenically inherited. Perhaps unsurprisingly, mating time is also to a degree a different phenotype in the two sexes and some of its genetic architecture may also therefore be different. Nevertheless, genes that make substantial contributions to the two characters are closely linked, albeit ultimately separable. As genomic resources continue to improve in *B. tryoni* and *B. neohumeralis* (e.g. a draft genome assembly [34], high-density linkage maps [35], transcriptomes [33]), it is now possible to use Genome Wide Association (GWA) analyses to tease apart the genetic architectures of the two characters, and of mating time in the two sexes, and to identify candidate genes and their functions which may be responsible for the phenotypes. High-resolution genetic mapping of mating time and callus colour loci (as well as genomic islands of differentiation) in *B. tryoni* and *B. neohumeralis* will become increasingly feasible once chromosomal level assemblies have been released (construction of second-generation chromosome assemblies is underway at the Oakeshott/Lee laboratory, CSIRO, Australia). Parallel genomic efforts in other tephritid species (e.g. *Ceratitis capitata* [36], *Z. cucurbitae* [37], *B. oleae* [38]) will also provide a useful syntenic comparison between their homologous chromosomal regions amongst these species. Nevertheless, implications of our findings for fundamental questions around species boundaries and for applied issues concerned with SIT are already clear.

The question of introgression between *B. tryoni* and *B. neohumeralis* was first posed several decades ago, along with speculation that it might be responsible for the rapid range expansion of *B. tryoni* over the last century [24]. However, it has generally been assumed that the difference in mating time − the only reproductive isolating mechanism known in this model system−would block interspecific gene flow. Against this, though, we have found that *B. neohumeralis* females do mate at dusk and *B. tryoni* males do not discriminate between them and their own conspecifics at that time, which implies bi-directional introgression is possible. These observations cast doubt on the effectiveness of mating time difference as a reproductive barrier. As noted earlier, there is currently some suggestive but no conclusive evidence for introgression in either direction. Observations of interspecific mating between *B. tryoni* and *B. neohumeralis* in nature have never been reported, although semi-field cage experiments have indicated a significant difference in their mating behaviours: *B. tryoni* mating pairs tend to aggregate at dusk but *B. neohumeralis* pairs do not [39]. We caution that our laboratory study might not be reflective of field conditions as fly densities in rearing cages were higher than typically experienced in field. It is likely that visitations to potential mating sites might vary temporally between species [39] and there might also be differences in mating site preference in addition to other elusive ecological pre-zygotic barriers that prevent natural introgression.

Notable in this context is the close genetic association we find between mating time and callus colour and the possibilities it raises of pleiotropic consequences in the hybrids and backcrosses. Unfortunately in this context little is currently known about the physiology underlying the callus colour differences but the persistence of an association through many generations of random mating suggests any detrimental pleiotropy could have significant effects on the fitness of backcrosses and their progeny and therefore the scope for introgression in the field. There is thus a possibility that the combined effects of the two diagnostic differences may generate a post-zygotic isolating mechanism. The fact that the two diagnostic differences are to a significant degree co-inherited is in fact reminiscent of long held speciation theories invoking co-adapted supergene complexes as barriers to hybridisation [40]. However, we also point out that there is yet no population genomic work to test whether hybridisation and introgression do occur in the field. Given its implications for theory, this is clearly now a priority for future work on the *B. neohumeralis* - *B. tryoni* model system.

Regarding the implications for SIT, our results show that mating time, particularly in *B. tryoni* males, is a polygenically inherited character and that it can change in relatively few generations under laboratory conditions. Important to note here is that while most studies of the phenomenon have found *B. tryoni*-like hybrids predominate over generations after an initial interspecific mating in the laboratory [18, 24], one study found the opposite result occurred under crowded conditions [23]. Mating time in some other bactrocerans have been found to evolve away from wild type timing during domestication in mass rearing facilities [e.g. [7, 8, 41, 42]]. Miyatake et al. (2002) and Economopoulos and Zervas (1982) were also able to induce significant shifts in mating time through selection for different developmental time in *Z. cucurbitae* and long-term domestication in *B. oleae*. Lighting conditions are known to play an important part in mating as well [16, 18, 43]; it is possible that particular lighting regimes could drive changes in mating time. While it is necessary for mass-rearing facilities (such as SIT factories) to implement highly efficient protocols that sustain high yields of sterile males, our results show that these protocols need to be developed with the preservation of wild-type mating behaviours in mind. This issue is particularly pertinent for *B. tryoni* given the increasingly important role SIT may play in its management.

## Conclusions

While we found both male and female *B. tryoni* prefer to mate at dusk and *B. neohumeralis* to mate several hours earlier during the day, *B. neohumeralis* females would mate at both day and dusk and *B. tryoni* males did not discriminate between females of the two species at that time. The results imply that interspecific hybridisation and introgression are possible, and that the mating time difference may be a weaker reproductive isolating barrier than once assumed. Major genes underlying the two traits are tightly linked although our recovery of recombinant phenotypes means that the two traits do have separable genetic architecture. However, the fact that strong selection affecting mating time was occurring in our hybrid populations and that this then affected callus colour frequencies for many generations means that pleiotropic effects may also come into play in determining the fate of any hybridisation events in the field. Our data also highlight the need for further work on possible changes in mating time profiles during domestication and mass rearing of SIT strains.

## Methods

### Insect provenance and husbandry

The *S06* strain of *B. tryoni* and the *neoC* strain of *B. neohumeralis* served as the reference parental strains for all phenotypic characterisation. S06 strain originated in Sydney (2006) [44] and while *neo*C was from Cairns (2006) [33] and have since been kept in laboratories at University of New South Wales (2006–2015) (approximately 60–70 generations) and CSIRO Black Mountain (2015 onwards). Larvae were reared on carrot diet [45] in the first 10 generations of the experiments described below before transitioning over to larval gel diet [46]. This was because the gel diet increased the productivity of the lines without affecting the parental mating time and callus colour phenotypes. Adults were reared in BugDorm cages (22.5 × 22.5 × 22.5 cm^3^) under constant temperature (25 °C) and relative humidity (70%) and a 13:11 light dark cycle provided with supply of sugar, yeast hydrolysate and water that were supplied ad libitum. The 13-h light cycle consisted of 1 h of < 100 lx of “dawn”, followed by 11 h of > 2000 lx “day” and 1 h of < 100 lx of “dusk”. Experiments commenced one generation after establishing in CSIRO Black Mountain (i.e., parental generation was approximately 61–71 generations since field collection).

### Construction and phenotypic characterisation of interspecific backcrosses

Six replicate mass hybrid crosses were set up between the parental (P) species (three replicates of 100 *B. tryoni* males × 100 *B. neohumeralis* females plus three replicates of the reciprocal) to produce the F1s (Fig. 1). F1 females from each of the six replicates were then backcrossed to *B. neohumeralis* males to generate six cohorts of backcross progeny (B1). Mating time and callus colour were scored in the P, F1 and B1 generations.

Mating time was scored in batches each consisting of 30 virgins of one sex from each tested line separately (parentals, F1 and B1) mixed with 30 virgins of the opposite sex from each of the two parental species (i.e., ~ 90 individuals per cage). The inclusion of both parental species was to ensure adequate supply of mates throughout the day cycle. Mating pairs were isolated and scored for callus colour on the same day. We tested a total of 120 parental males, 120 parental females, 240 F1 males, 240 F1 females, 2460 B1 males and 2280 B1 females. We started observations for approximately half of all the males and females at dusk and the other half in the morning, akin to the day/dusk filtering in Meats et al. (2003). Under this filtering regime, day filtered flies had not responded to an opportunity to mate during the day previous to their test at dusk, whereas the dusk filtered flies had not responded to an opportunity to mate at the dusk prior to their test during the day.

Callus colour was inspected under a dissecting microscope (10x magnification) and binned into one of five ordinal classes according to the relative amount of yellow in the callus, following Pike et al. [28]: Class ‘bbbb” was fully brown, a *B. neohumeralis*-like phenotype, “bbbY” was mostly brown with between 5 and 25% yellow, “bbYY” was about 50% yellow,” bYYY” was mostly yellow (> 70%) and” YYYY” was completely yellow, a *B. tryoni*-like phenotype.

### Mating time selection in advanced generation hybrid populations

We maintained three of the six replicates of F1 hybrids generated for the backcross experiment for an additional 24 generations. Six hundred F25 males (200 per replicate population) were placed individually with one female of each parental type in individual plastic cups. Unlike the backcross experiment, we started mating time observations during the day. Mating pairs were not removed from the cups. Mating events were recorded over two consecutive days. We classified all those males that mated at least once as follows: “early mating” males mated at least 1 h before dusk at least once and did not mate at any other time; “dusk mating” males mated at dusk at least once and did not mate at any other time; and “other” males either mated within 1 h prior to dusk at least once or re-mated outside their original mating time.

The selection procedure involved mating “early mating” males individually to virgin F25 females of the corresponding replicate to establish 18 isofemale “early mating” lines, and mass mating “dusk mating” males to females from the corresponding replicate to establish three replicate mass bred “dusk mating” lines. We then applied the same bioassay and selection procedures to all 18 early mating isofemale lines at F26. The offspring of the F25 “dusk mating” lines were also scored for mating time and callus colour, but we discontinued the lines at that point because there was no strong shift in mating time.

Three early mating lines that had sufficient progeny were further mass bred for eight generations without selection. Two of these three early mating sublines were isofemale lines generated from replicate cage 1 at F25 (1_1 and 1_5) and the third was a pool of isofemale lines from replicate cage 2 at F25. These sublines were rescored for mating time and callus colour at F35 (720 individuals tested, half of each sex, 240 per replicate line), again using the same protocol as for F25.

To estimate recombinant frequencies, we scored callus colour in subsets of the F25 and F35 “dusk mating” males that had mated at dusk on both days of testing. We considered these subsets of repeat dusk mating males to be the most reliably phenotyped for both characters.

### Statistical analyses

The variables in the backcross experiment included maternal background, start time of mating observation, biological replicates, species of the parentals, the observed mating time and callus colour. We simplified the analysis by grouping the mating time observations into three major categories, day mating (Day-neo), intermediate and dusk mating (Dusk-try), where Day-neo is the mating time of *B. neohumeralis*, Dusk-try is the mating time of *B. tryoni* and intermediate is determined to be the period of up to 3 h before onset of dusk. We approached the data conservatively by only preserving the dependent variables that had distinct patterns while merging the data for those variables that had little change in trend for mating time and callus colour. For all backcross data (parental, F1 and B1), we visualised the proportion of each mating time class and callus colour class by sex and parental species mated unless no information was obtained for those variables. Replicates were used to generate confidence interval. To show if mating time and callus colour traits were linked, we performed a contingency test to test whether the two traits were independent at B1.

In the advanced generation hybrid population, we presented data for male mating time only but included callus colour for all flies. We analysed raw mating time rather than grouping them into the simplified classes used for the backcrossed experiment as there was far less variability. We performed the contingency test again on F25 males to show whether callus colour was statistically independent of mating time. To show that the selection for early mating resulted in variable selection responses, we ran a non-parametric Kruskal-Wallis test where mating time was the dependent variable and isofemale line was the independent variable. For all other comparisons between generations for mating time and callus colour, we performed multiple pairwise comparisons using Mann-Whitney *U* or unpaired *t-*tests corrected for multiple comparisons with the family-wise Holm-Bonferroni method. To test for change in proportion between F25 and each selected line, we employed the proportion test with Yates continuity correction to test for differences in proportions/percentages.

All analyses and plots were run/generated in R [47]. We used the following packages: ‘grid’, ‘dplyr’ [48], ‘tidyr’ [49], ‘ggplot2’ [50], ‘cowplot’ [51], ‘ggpubr’ [52], ‘stringr’ [53] and ‘rcompanion’ [54]. Package ‘grid’ allowed rendering of images into plots, ‘dplyr’ was used to synchronised two or more separate dataset, ‘tidyr’ allowed us to use the function ‘complete’ to output all possible combination of factors including missing combinations, ‘ggplot2’ and ‘cowplot’ was necessary for plotting the data, while we used ‘ggpubr’ for calculation and graphical representation of multiple comparison tests, ‘stringr’ for manipulating characters and ‘rcompanion’ for a function to calculate median confidence interval. Several specialised scripts were written to synchronise the format of the data of different experiments performed over the four-year period.

## Data Availability

All data generated or analysed during this study including statistical analysis codes to regenerate the results are available from github via https://github.com/nilpy/BactroceraMating-Callus

## Abbreviations

P: Is for parental
F1: Is the offspring of hybridisation cross between the two parentals
B1: In this study is the offspring of F1 female x *B. neohumeralis* male.
